# Orexin Decreases *Aromatase* Gene Expression in The Hypothalamus of Androgenized Female Rats

**DOI:** 10.22074/ijfs.2016.4909

**Published:** 2016-06-01

**Authors:** Maliheh Salimi, Zahra Alishah, Homayoun Khazali, Fariba Mahmoudi

**Affiliations:** 1 Department of Physiology, Faculty of Biological Sciences, Shahid Beheshti University, Tehran, Iran; 2 Department of Biology, Facualty of Basic Sciences, University of Mohaghegh Ardabili, Ardabil, Iran

**Keywords:** Orexin, Cyp19, Female Rats

## Abstract

**Background:**

Orexin is a hypothalamic orexigenic neuropeptide, which third cerebral injection of it mainly exerts inhibitory effects on reproductive functions. It increases significantly the *Aromatase* (*Cyp19*) gene expression in the hypothalamus of male rats. Aromatase is an enzyme which converts androgens to estradiol in the hypothalamus of rats. Prenatal or neonatal exposure of females to testosterone masculinizes the pattern of *Cyp19* mRNA levels in adulthood. In the present study the effects of central injections of orexin-A on hypothalamic *Cyp19* gene expression of adult female rats were investigated, while they had been androgenized on third day of postnatal life.

**Materials and Methods:**

In this experimental study, twenty female Wistar rats received subcutaneous injections of testosterone propionate (50 µg/100 µl) on their third day of postnatal life. Adult androgenized rats weighing 180-220 g, received either 3 µl saline or one of 2, 4 or 8 µg/3 µl concentration of orexin via third cerebral ventricle. Five non-androgenized rats, as control group, received intra cerebral ventricle (ICV) injection of 3 µl saline. The hypothalamuses were dissected out and mean *Cyp19* mRNA levels were determined by semi-quantitative real time-polymerase chain reaction (PCR) method. Data were analyzed by unpaired t test and one-way ANOVA using SPSS software, version 16.

**Results:**

Mean relative *Cyp19* mRNA level was significantly increased in the hypothalamus of androgenized compared to non-androgenized female rats. Central injec- tions of 2, 4 or 8 µg/3 µl orexin decreased significantly the hypothalamic *Cyp19* mRNA level of androgenized rats compared to androgenized-control groups.

**Conclusion:**

The results suggested that the orexin may exert inhibitory effects on the gene expression of *Cyp19* in the hypothalamus of neonatal androgenized female rats in adulthood.

## Introduction

In mammals, a complex network of central and peripheral signals controls the hypothalamuspituitary-gonadal (HPG) axis. Among the peptides involved in the control of energy balance and reproduction, orexin neuropeptides are important factors for regulation of the reproductive axis. Orexin-A is a 33 amino-acids orexigenic neuropeptide (1, 2). It is mainly synthesized in the lateral hypothalamus and the fibers project to the hypothalamic nucleui to regulate the reproductive functions (3, 5). The mechanism, whereby orexin affects HPG axis is not completely clear yet and both stimulatory and inhibitory effects of orexin-A or -B were observed on pulse frequency and pulsatile secretion of gonadotropin-releasing hormone/luteinizing hormone (GnRH/LH) release in female rats (6, 12). Aromatase cytochrome P450 is an enzyme coded by *Cyp19* gene (also known as *Cyp19A1* or *P-450AROM*). While the highest activity is observed in the hypothalamic nuclei including median preoptic area, this enzyme converts androgens (e.g. testosterone) to estradiol in peripheral tissues and brain (13, 15). Testosterone is an important regulator of the *Cyp19* gene expression in the hypothalamus of rats (13). Owing to distinct circulating androgen levels in different sexes, the expression of *Cyp19* gene in the male rat hypothalamus is greater than in females (13, 15). It has been demonstrated that prenatal or neonatal females, receiving exogenous injection of testosterone propionate (TP) during some crucial developmental stages, could partially exhibit infertility in adulthood due to the *Cyp19* gene expression increase (16, 19). Considering that elevation of androgen could lead to many abnormalities, like hyperandrogenic disorders and anovulation, study the pathophysiological effects of this hormone aberration appears to be very critical (18). Investigations show that aromatase could regulate not only the masculine sexual behaviour in males, but also the cyclic ovulatory LH surge in females (13). Curiously, it has been demonstrated while aromatase applies a prohibition effect on the ovulation procedure by blocking gonadotropin surges, aromatase inhibitors could exert a stimulatory effect on this procedure (13, 20). It has been reported that hypothalamic interneurons-including neuropeptide Y (NPY), pre-opiomelanocortin (POMC) or ghrelinmay play a role in mediating the inhibitory effects of orexin on HPG axis (21, 23). We have previously shown that central injection of orexin significantly increased the *Cyp19* gene expression and estradiol hormone levels in the hypothamus of male rats (24). The purpose of present study was to investigate the effects of orexin central injection on hypothalamic *Cyp19* gene expression levels in androgenic rat model, Materials and Methods Animals In the present experimental study, twenty neonatal female Wistar rats (provided by Neurophysiology Research Center of Shahid Beheshti University, Tehran, Iran) received subcutaneous injection of TP (50 µg/100 µl) on the third day of postnatal life, as with previous studies (25, 32). Also, five non-androgenized female rats were used as control group. Control and androgenized pups were housed with their mothers in cages under conventional control of temperature (22 ± 2°C) and light (12 hours light/dark cycle, light on 07:00 hours). Animals had free access to food and water all the time. All animal procedures were performed in accordance with ethical committee of Shahid Beheshti University. The procedures was designed consistent with previous investigations whereby the injection of TP single dose into neonatal female rats led to persistent adulthood infertility, 100 days after birth (25, 32). 

## Materials and Methods

### Animals

In the present experimental study, twenty neonatal female Wistar rats (provided by Neurophysiology Research Center of Shahid Beheshti University, Tehran, Iran) received subcutaneous injection of TP (50 µg/100 µl) on the third day of postnatal life, as with previous studies (25-32). Also, five non-androgenized female rats were used as control group. Control and androgenized pups were housed with their mothers in cages under conventional control of temperature (22 ± 2°C) and light (12 hours light/dark cycle, light on 07:00 hours). Animals had free access to food and water all the time. All animal procedures were performed in accordance with ethical committee of Shahid Beheshti University. The procedures was designed consistent with previous investigations whereby the injection of TP single dose into neonatal female rats led to persistent adulthood infertility, 100 days after birth (25- 32).

### Intra cerebral ventricle cannulation and injection

Animal surgery procedures and handling were carried out as previously described (33). Adult control and androgenized rats with 100 days of age (26,32) and 180-220 g body-weight (BW) were anesthetized using intraperitoneal (IP) injection of a ketamine and xylezine mixture (ketamine 80 mg/kg BW+xylezine 10 mg/kg BW). For central injections, a 22gauge stainless cannula was implanted into the third cerebral ventricle according to coordinates of Paxinos and Watson Atlas ( [anterior-psterior (AP)=-2.3, mid line (ML)=0.0, dorsal-ventricle (DV)=6.5]. The cannula was secured to the skull with three stainless steel screws and dental cement. The animals were kept in individual cages. After one week recovery period, twenty androgenized rats in four groups (five rats in each group) received either 3 µl saline or one of the 2, 4 or 8 µg/3 µl orexin-A via third cerebral ventricle. 

Five non-androgenized rats, as control group, received ICV injection of 3 µl saline in estrous phase of estrous cycle at the 100 days of age. The appropriate doses of orexin were selected with regards to our previous studies, implicating on the stimulatory and inhibitory effects of orexin on Cyp19 gene expression in the hypothalamus and HPG axis of male rats (12,16). Orexin-A (Ana spec Co., USA) was dissolved in saline and injected Intra-cerebroventricularly by a 27gauge stainless steel injector (protruded 0.5 mm beyond the cannula), connected to Hamilton microsyringe by polyethylene (PE-20) tubing between 09:00 and 10:00 a.m. For subcutaneous injection, TP was dissolved in olive oil and injected by an insulin syringe. At the end of the experiment, these rats were anesthetized, sacrificed by decapitation and subsequently the brains were quickly collected. The hypothalamuses were dissected out as previously described (34). The samples were immediately frozen in liquid nitrogen and stored at -80°C. 

### RNA isolation and semi-quantitative reverse transcription-polymerase chain reaction

Total RNA was isolated from individual frozen hypothalamus. Total RNA was extracted using pureZol RNA isolation reagent according to manufacturer instruction (BioRAD, USA). The quantity of each RNA sample was performed by measuring absorbance at 260 nm. Regarding that β-Actin (Actb) transcription is consistently expressed within different tissues, including brain, it was considered as housekeeping gene to normalize the other gene mRNA expression levels, using semi-quantitative reverse transcriptionpolymerase chain reaction (RT-PCR) technique. For that, the first cDNA strand was synthesized from 5 μg of total RNA according to manufacturer instruction (RT-PCR kit, vivantis, Malaysia). Subsequently, Cyp19 and β-Actin genes fragment were respectively amplified on 34 cycles (94°C for 30 seconds, 61°C for 30 seconds, 72°C for 30 seconds) and 35 cycles (94°C for 30 seconds, 58°C for 30 seconds, 72°C for 30 seconds) at a final volume of 50 μl containing cDNA template (2 µl), 10X PCR buffer (5 µl), 50 mM MgCl_2_ (1.5 µl), 10 mM dNTP Mix (1 µl), 100 µM sense and antisense primers (1 µl of each one) and 500 U Taq-DNA Polymerase (0.5 µl) as well as sterile water (38 µl) according to manufacturer instruction (PCR kit, vivantis, Malaysia). PCR amplification produced a 511 base pairs (bp) fragment using β-Actin-F: 5′-GAAATCGTGCGTGACATTAAG-3′ and β-Actin-R: 5′-GCTAGAAGCATTTGCGGTGGA-3′ primers (35,36), or a 289 bp fragment using Cyp19-F: 5′-GCTTCTCATCGCAGAGTATCCGGCA-3′ and Cyp19-R: 5′AGGGTAAATTCATTGGGCTTGG-3′ primers (37). The RT-PCR products were analysed by 1% agarose gel electrophoresis. Band intensities were compared by imaging safe view staining and quantified using ImageJ software program. 

### Statistical analysis

Differences between androgenized and non-androgenized control groups were assessed using student unpaired t test. Significant differences of orexintreated groups were determined by one-way ANOVA followed by post hoc Dunnet test, using SPSS software version 16. A P value of 0.05 was considered as significant threshold. Data presented as the mean value with SEM of independent experiments. 

### Results

**Fig.1:**
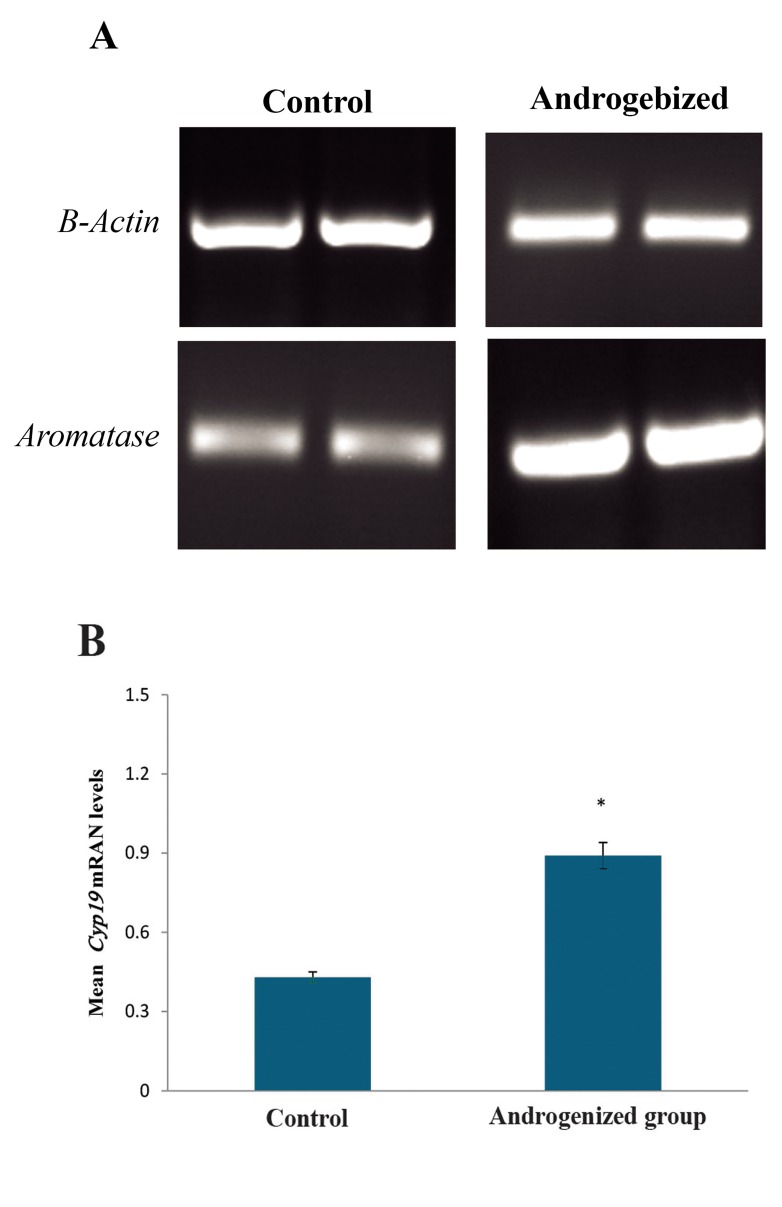
Mean relative Cyp19 mRNA levels in the hypothalamus of androgenized female rats compared to control group. A. Representative agarose gel electrophoresis of products corresponding to β-Actin and Cyp19, amplified by RTPCR method and B. Cyp19 mRNA levels (mean ± SD) in each group (n=5) were semi-quantitatively determined by ImageJ software. The cDNA amplified from β-Actin mRNA was to normalize corresponding Cyp19 results. *; P<0.05 and RT-PCR; Reverse transcription polymerase chain reaction.

To study the effect of Cyp19, transcription of this gene was semi-quantitatively compared between hypothalamus of the five androgenizedand five control-saline-treated rats. Qualitative results showed that relative Cyp19 mRNA levels in hypothalamus of the androgenized rats are significantly higher than controls (Fig .1A). A further analysis was applied to relatively determine the semi-quantitative levels of Cyp19 mRNA using qualitative results densitometry scanning. Findings demonstrated that mean hypothalamic Cyp19 mRNA levels were significantly increased in the hypothalamus of androgenized saline-treated rats to 83%, in comparison with control salinetreated rats (Fig .1B). 

**Fig.2:**
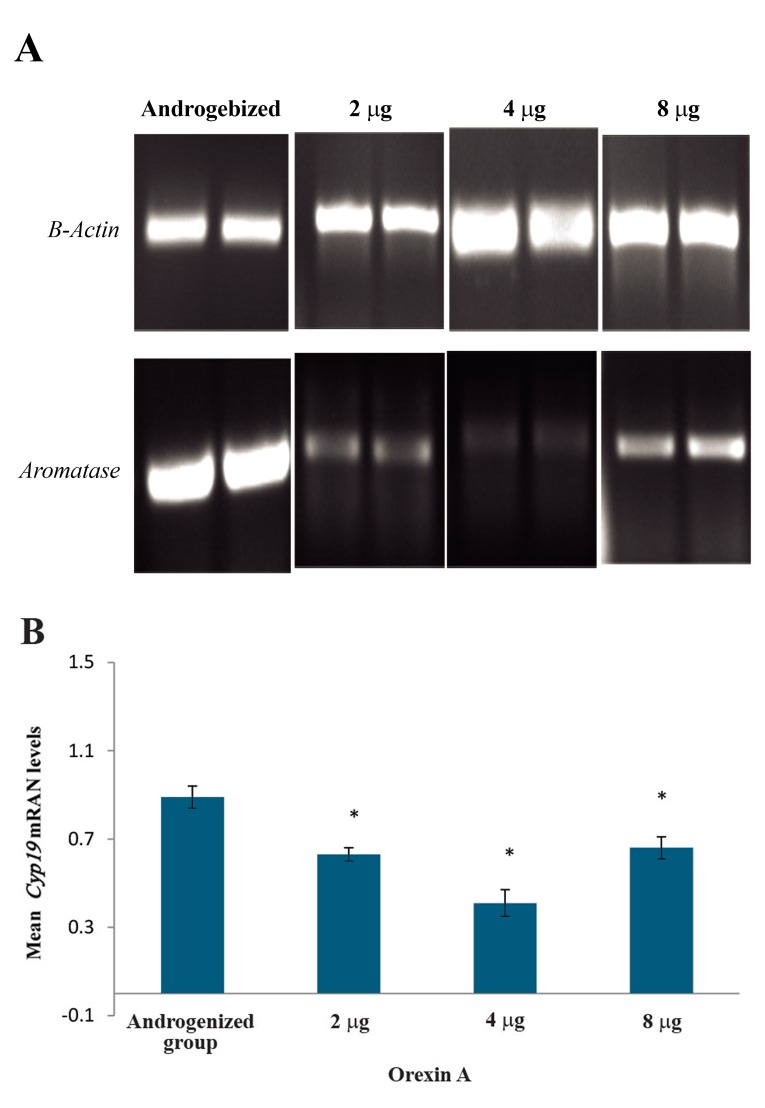
The effects of central injections of 2, 4 or 8 µg orexin-A on Cyp19 mRNA levels in the hypothalamus of androgenized female rats compared to androgenized-saline group. A. Representative agarose gel electrophoresis of products corresponding to β-Actin and Cyp19, amplified by RT-PCR method and B. Relative mean Cyp19 mRNA level (mean ± SD) in each group (n=5) was semi-quantitatively determined by ImageJ software. The cDNA amplified from β-Actin mRNA was to normalize corresponding Cyp19 results. *; P<0.05 and RT-PCR; Reverse transcription polymerase chain reaction.

We subsequently investigated the effect of different orexin-A concentrations on Cyp19 mRNA expression level in hypotalamos of the presented groups. In terms of the quality, data analyses of central injection showed that injection of 2, 4 or 8 µg orexin-A led to reduction of hypothalamic Cyp19 mRNA levels compared to androgenized control group (Fig .2A). Figure 2B provides a semi-quantitative analysis of the data determined by densitometry scanning (obtained from 5 animals used in experiments for each treatment). These results also showed that injections of 2, 4 or 8 µg orexin-A decreased hypothalamic Cyp19 mRNA levels by 20, 48 or 16% compared to androgenized-saline group. In all three groups, this decrease in the mean Cyp19 mRNA levels was statistically significant compared to androgenized-saline group (Fig .2B). 

## Discussion

The result of the present study showed that relative mean Cyp19 gene expression was significantly increased in the hypothalamus of neonatal androgenized female rats (with the age of 100 days of life) compared to non-androgenized adult control rats (in estrous phase of estrous cycle, with the age of 100 days of life). This result is consistent with the previous studies which reported that neonatal exposure of female rats to testosterone masculinizes the pattern of hypothalamic Cyp19 gene expressions in adulthood (16,19,26,32). It is well known that existence of androgens during critical differentiation period of brain sexual regions (late gestation and continues into ten days of postnatal life) can permanently alter the gender-specific capacity for aromatization in the hypothalamus (38,39). Previous studies showed that high level of Cyp19 gene expression in the hypothalamus of androgenized female rats may be a possible mechanism whereby androgen induces sterility and lack of cyclic ovulatory discharge of LH in adulthood. 

Orexin is a hypothalamic neuropeptide which exerts mostly inhibitory effects on reproductive axis (6,12,40). It has interestingly been established that aromatase exert an inhibitory effect on ovulation via inhibiting gonadotropin surges (13,20). So that aromatase levels were low during estrous phase of estrous cycle (32). In the present study, the potential effects of central injection of orexin were investigated on Cyp19 gene expression in androgenized female rats. The results showed that hypothalamic Cyp19 mRNA levels were significantly decreased in orexin-treated androgenized rats compared to androgenized control group. In the present study, for the first time we determined the effects of orexin on Cyp19 gene expressions in the hypothalamus of androgenized female rats. So far, there is not any report to indicate the exact mechanism leading to reducing Cyp19 gene expression in adult neonatal androgenized female rats, upon induction of orexin. Never the less, it has been revealed that the levels of Cyp19 gene expression in the hypothalamus of TP-treated perinatal or neonatal females are not significantly different from those in the hypothalamus of normal male rats (38,39). We have also previously reported that central injection of orexin significantly increased the mean Cyp19 mRNA levels and mean estradiol concentrations in the hypothalamus of wild type male rats (24). In regard to these results, we initially estimated to observe similar stimulatory effects of orexin on Cyp19 gene expression in neonatal androgenized female rats. Although, the reason of this controversy between the later female and wild type male rats is not clear. One possible difference between the Cyp19 gene expression patterns in these groups of rats may be due to blood testosterone levels in adulthood. Roselli and Klosterman (14) reported that exposure of the brain to steroid hormones appears to be necessary for sexual differentiation of Cyp19 expression during prenatal life, although gonadal hormones might also be able to exert additional effects during puberty and adulthood. On the other hand, it is possible that plasma testosterone concentration in adult male rats affects the orexin influence on Cyp19 mRNA level in a different manner compared to androgenized adult female rats. Although further studies are required to better understand the exact effects of orexin on Cyp19 gene expression pattern in female rats, a side specific effect of orexin on GnRH/ LH release could be another cause of obtaining present results (11). 

It has been shown that the brain areas -involved in the controlling HPG axis including rostral preoptic area (rPOA), medial POA (mPOA) and arcuate nucleus/median eminence (ARC/ME)is innervated by orexin neurons, leading to effect differently on the LH release in female rats. So that, orexin enhances LH levels after injection into the rPOA, while it inhibits LH release after injection into the mPOA or ARC/ME (11). In this study, the effect of orexin on hypothalamic Cyp19 gene expression was determined by injection into the third cerebral ventricular injection of the androgenized female rats. Like LH release, injection of orexin into HPG axis specific nuclei might differently affect Cyp19 gene expression. Therefore, to discuss the exact effect of orexin on Cyp19 gene expression in neonatal androgenized adult females, it is recommended to investigate the effect of orexin on Cyp19 gene expression of rPOA, mPOA or ARC. 

## Conclusion

This paper demonstrated that mean Cyp19 mRNA level was significantly increased in the hypothalamus of three days-old androgenized control rats compared to non-androgenized control ones. As a novel finding, it was also reported that injection of orexin significantly decreased the mean hypothalamic Cyp19 mRNA level in androgenized female rats compared to androgenized control group. 
